# The More the Worse: the Grade of Noise-Induced Hearing Loss Associates with the Severity of Tinnitus

**DOI:** 10.3390/ijerph7083071

**Published:** 2010-08-04

**Authors:** Birgit Mazurek, Heidi Olze, Heidemarie Haupt, Agnieszka J. Szczepek

**Affiliations:** 1 Molecular Biology Research Laboratory and Tinnitus Center, Department of Otorhinolaryngology CCM, Charité - Universitätsmedizin Berlin, Charitéplatz 1, 10117 Berlin, Germany; E-Mails: heidemarie.haupt@charite.de (H.H.); agnes.szczepek@charite.de (A.J.S.); 2 Department of Otorhinolaryngology CVK, Charité - Universitätsmedizin Berlin, Augustenburger Platz 1, 13353 Berlin, Germany; E-Mail: heidi.olze@charite.de (H.O.)

**Keywords:** noise, noise-induced hearing loss, tinnitus, quality of life

## Abstract

Tinnitus disturbs lives and negatively affects the quality of life of about 2% of the adult world population. Research has shown that the main cause of tinnitus is hearing loss. To analyze a possible association of the degree of hearing loss with the severity of tinnitus, we have performed a retrospective study using admission data on 531 patients suffering from chronic tinnitus. We have found that 83% of our tinnitus patients had a high frequency hearing loss corresponding to a noise-induced hearing loss (NIHL). There was a significant correlation between the mean hearing loss and the tinnitus loudness (p < 0.0001). Interestingly, patients suffering from decompensated chronic tinnitus had a greater degree of hearing loss than the patients with compensated form of tinnitus. In addition, we demonstrate that the degree of hearing loss positively correlates with the two subscales (“intrusiveness” and “auditory perceptional difficulties”) of the Tinnitus Questionnaire. Our retrospective study provides indirect evidence supporting the hypothesis that the degree of noise-induced hearing loss influences the severity of tinnitus.

## 1. Introduction

Tinnitus is a perception of sound without an external source. This perception can be induced by various dysfunctions on several levels of the peripheral or central auditory pathway [1]. Regardless of the original cause, all patients complain of hearing a tinnitus tone on either one (unilateral) or both sides (bilateral) of the head or ears. Depending on the case, tinnitus tone may have low, medium or high frequency and be either relatively quiet (0–3 dB), going up to relatively loud (more than 16 dB). Tinnitus may take acute (up to 3 months), sub-acute (4–12 months) or a chronic turn (longer than a year). Regarding the level of disturbance, tinnitus can be classified as compensated (low-level distress) or decompensated (high-level distress) [2]. The major problem in patients with decompensated tinnitus is sleep interference, because the tinnitus tone keeps the patients awake. Other diseases that follow include depression, a variety of phobias, anxiety disorders, problems with concentration and in extreme cases—suicide. In other words, decompensated tinnitus seriously reduces the quality of life. Approximately 30 per 100 adults experience tinnitus, whereas about 1–5 persons per 100 suffer from tinnitus and seek medical help [3,4]. In the Western world, tinnitus has a big economic impact [5].

The onset of tinnitus can have various basis such as neurologic, traumatic, infectious or drug-related [6], however, the major cause of tinnitus is a hearing loss [5]. Hearing loss is usually caused by the aging process (presbycusis) or by the overexposure to noise (noise-induced hearing loss, NIHL). Occupational noise, together with environmental noise pollution, are two major factors contributing to the NIHL [7]. Newly emerging NIHL victims are adolescents who inappropriately use MP3 or MP3-like personal players (too long/too loud, using earphone-insert type headphones) [8].

Between 57% and 76% of tinnitus patients were shown to have NIHL [9–11]. These, and a lot of other data, strongly indicate coexistence of both hearing dysfunctions. Based on the above data we put forward a hypothesis that the degree of hearing loss could negatively influence the severity of tinnitus.

To test our hypothesis we used a retrospective study using data acquired from 531 tinnitus patients. This data were randomly collected on the admission of patients who reported to the day ward of Tinnitus Center at the Charité - Universitätsmedizin in Berlin between January 2008 and March 2010. We have analyzed general audiometric and tinnitus-oriented psychometric parameters.

## 2. Experimental Section

### 2.1. Participants

We have obtained the data from 531 tinnitus patients (sub-acute and chronic) admitted to a 7-day multimodal tinnitus therapy. The data were collected on the admission day. The data were collected between January 2008 and March 2010. Of 531 tinnitus patients, 280 were women and 251 men. The mean age of the patients was 49 years (SD = 13, min 16, max 79).

### 2.2. Audiometry

Audiometric tests were performed using routine pure tone audiogram (0.5–8 kHz). In addition, tinnitus frequency (kHz) and tinnitus loudness (dB HL) were matched for each patient.

### 2.3. Psychometry (Tinnitus Questionnaire)

Tinnitus-related distress was assessed using validated standard German version of the tinnitus questionnaire (TQ). The TQ is composed of 52 items allocated to six scales: emotional distress, cognitive distress, intrusiveness, auditory perceptual difficulties, sleep disturbances and somatic complaints [12]. The severity level of tinnitus was assigned as “compensated” (low-level distress) with TQ score ≤ 46 points and “decompensated” (high-level distress) with TQ score > 46 points.

### 2.4. Statistics

Groups were compared using the Mann-Whitney U-test. Spearman's rank correlation was used to test the statistical relationship between hearing loss and tinnitus parameters or tinnitus severity. P < 0.05 was the level of significance; the confidence intervals were 95% (Statistica 7.1, StatSoft).

## 3. Results

### 3.1. Predominant Number of Patients with Chronic Tinnitus Has a Hearing Loss

**Figure 1.**
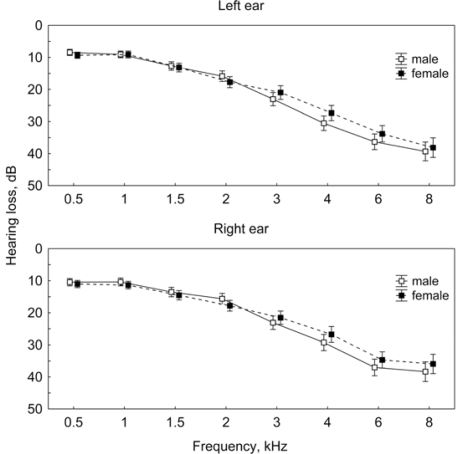
Pure tone audiogram measured on the left and right ear of the male and female tinnitus patients with hearing loss in the upper frequency range. Given are the means ± confidence intervals.

Of all 531 tinnitus patients tested, 441 (83%; 220 men and 221 women) had a hearing loss in the upper frequencies (Figure 1), corresponding to a pattern found in the NIHL [13]. There were no significant differences in the hearing loss between the left and the right ear and between male and female patients in this group. Of remaining 90 patients, 84 had pantonal hearing loss and six persons heard normally. The mean age of tinnitus patients with NIHL-like audiogram was 50 years (SD = 13, min 16, max 79), with patients most frequently ranging between 40–60 years of age.

### 3.2. Tinnitus Characteristics in Patients with Hearing Loss

Of 441 tinnitus patients with NIHL-like audiogram, 319 patients had pure tone tinnitus whereas 122 patients had noise tinnitus. Forty-seven percent had unilateral tinnitus and 53% had bilateral tinnitus.

**Figure 2.**
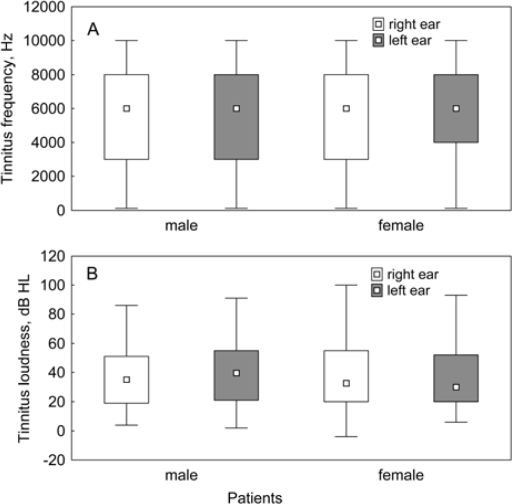
Tinnitus frequency (A) and tinnitus loudness (B) measured in the right and left ear of the male and female tinnitus patients with hearing loss. Given are the medians, percentiles (25–75%) and min-max values.

The median value of the tinnitus frequency measured on the left or right side of patients with pure tone tinnitus was 6.0 kHz (Figure 2A). There were no differences between female and male patients. The median value of the tinnitus loudness was 34.0 dB HL on the right ear and 37.0 dB HL on the left ear. There were no differences between left and right ear (Figure 2B) or between men and women.

The mean tinnitus-related distress of the 441 NIHL-like audiogram /tinnitus patients was 34.2 points (SD = 16.3) as measured using the TQ. The values did not differ between female and male patients except for the subscale “somatic complaints” with woman having significantly greater scores than the male patients (Table 1). Of the 441 patients, 335 patients suffered from compensated tinnitus (TQ ≤ 46 points) and 106 patients suffered from decompensated tinnitus (TQ > 46 points).

### 3.3 The Degree of Hearing Loss Correlates with the Loudness of Tinnitus

We found a close correlation between the mean hearing loss and the tinnitus loudness on the right and left sides (r = 0.67 and r = 0.60, respectively; p < 0.0001). Within single frequencies, the closest correlation emerged between the tinnitus loudness and the hearing loss measured at 6 kHz (r = 0.68 and 0.62, right and left side, respectively).

### 3.4 The Degree of Hearing Loss Correlates with Some of the Psychometric Parameters Measured by TQ

**Figure 3.**
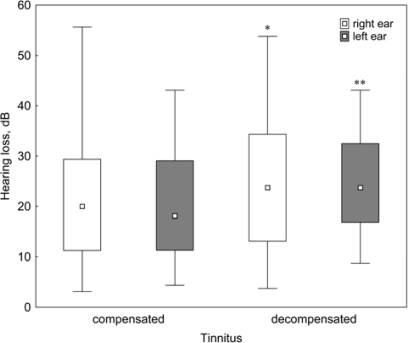
Hearing loss measured in the right and left ears of patients with compensated and decompensated tinnitus. Given are the medians, percentiles (25–75%) and min-max vales. *p < 0.05 and **p < 0.01 *vs*. compensated tinnitus.

Hearing loss was of significantly higher degree in patients with decompensated tinnitus than in patients with compensated tinnitus (Figure 3). This means that tinnitus patients with greater hearing loss experience higher tinnitus-related distress. Although the correlation between the total score of TQ and the mean hearing loss on the right and left sides was weak, nevertheless it was statistically significant (r = 0.17, p < 0.01).

During the analyses of separate TQ subscales, we found a correlation between the hearing loss and the TQ subscales “intrusiveness” and “auditory perceptual difficulties” (r = 0.33 and r = 0.24, p < 0.0001). We found no correlation between tinnitus frequency or loudness and distress caused by tinnitus.

### 3.5. Influence of Age on the Psychometric Scores

**Figure 4.**
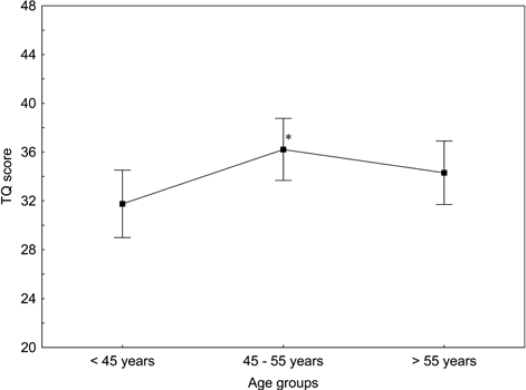
TQ scores measured in the three age groups of tinnitus patients with the hearing loss. Given are the means ± confidence intervals *p < 0.05 *vs*. patients < 45 years.

Our recent study [14] revealed that the age of patients may influence their TQ values. Consequently, in the present study, we have subdivided the patients into three age groups and analyzed their TQ scores as a function of age. The TQ scores increased with age in the group younger that 55 and decreased with age in patients older than 55 (Figure 4), whereas the hearing loss increased continuously with age (r = 0.57 and r = 0.59, p < 0.0001). The degree of hearing loss in the patients younger than 55 correlated weakly with the mean TQ scores (r = 0.22, n = 234, p < 0.001).

### 3.6. Influence of Tinnitus Lateralization on the Psychometric Scores

**Figure 5.**
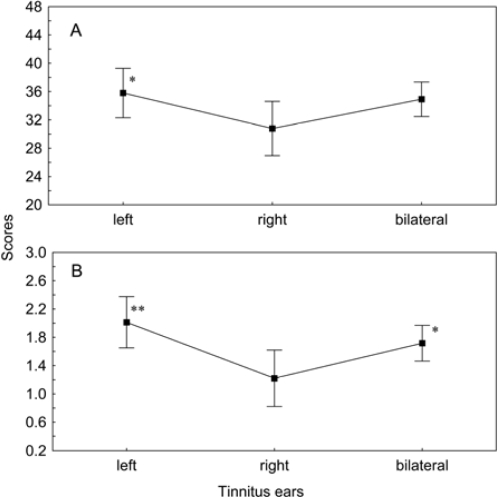
Total TQ scores (A) and scores of the subscale “somatic complaints” (B) measured in patients with left-side, right-side or bilateral tinnitus. Given are the means ± confidence intervals. *p < 0.05 and **p < 0.01 *vs*. right-side tinnitus.

We compared the tinnitus-related distress between the patients with left-sided, right-sided and bilateral tinnitus. Despite matching values of the hearing loss, tinnitus frequency and tinnitus loudness, the TQ score was significantly higher in patients who had tinnitus on their left side as compared to the right-sided tinnitus (p < 0.05) (Figure 5a). Within the subscales, there was a pronounced difference in “somatic complaints” between the left-sided and right-sided tinnitus (p < 0.01) and between right-sided and bilateral tinnitus (p < 0.05) (Figure 5b). There were no remarkable differences between male and female patients.

## 4. Discussion

Our present work was aimed at analyzing the association between noise, the grade of the noise-induced hearing loss and the severity of tinnitus. Our results partially corroborate the findings of others by demonstrating a strong bond between the noise-induced hearing loss and tinnitus [10]. In fact, we have shown that 83% of patients with chronic and sub-acute forms of tinnitus have a high-frequency hearing loss, which is a substantially higher percentage than the 57% reported by Savastano or the 63% reported by Martines *et al.* [9]. The sample size in all studies was roughly similar, as were the age and gender distribution. However, there was a major difference in the duration of tinnitus between our and the other studies. In the Savastano study, 49% of tinnitus patients had suffered from tinnitus for a time shorter than one year – a group classified as acute and sub-acute tinnitus. In the Martines *et al.* study, there were 38% of patients with acute (duration of less than three months) and 36% of patients with sub-acute tinnitus (between four and twelve months). Of our patients, none suffered from acute tinnitus. Based on the above evidence, one is tempted to cautiously speculate that the tinnitus associated with hearing loss has a greater chance of chronification than the tinnitus without hearing loss. Obviously, more studies are needed to validate this observation. In the other study, Attias and colleagues have indentified a subgroup of patients with tinnitus, who were actively looking for a remedy. This group was called “Tinnitus Seeking Help Group” (n = 355) [11]. Intriguingly, 100% of the “Tinnitus Seeking Help Group” suffered from the NIHL. This observation supports our records and suggests similarities between the NIHL, “Tinnitus Seeking Help Group” and our patients, who also were actively looking for help.

We found a significant correlation between the mean hearing loss and the tinnitus loudness (p < 0.0001).The fact that the loudness of tinnitus is increased in patients with coexisting hearing loss has been already known [15], but the observation of the dependency of tinnitus loudness on the severity of the hearing loss is a new one. Our study gives a base for the explanation of how the degree of noise-induced hearing loss indirectly affects the severity of tinnitus by influencing negatively the loudness of tinnitus. In agreement with this, the degree of hearing loss correlated positively with two subscales (“intrusiveness” and “auditory perceptional difficulties”) of the Tinnitus Questionnaire. These particular items of TQ regard the ability to communicate, which is an integral part of the quality of life. There is a known negative association between tinnitus distress and the quality of life. The most affected areas of life are social activities like communication and social life. With the increased distress of tinnitus, patients withdraw from their normal social life and avoid contacts with others, consistent with the decompensated form of tinnitus. Interestingly, we found that the patients suffering with decompensated tinnitus had a greater degree of hearing loss than the patients with compensated tinnitus.

Noise pollution and occupational noise were demonstrated to induce asymmetric hearing loss, with the left side being affected more than the right one [13]. Although our analyses demonstrated no statistically significant differences in the hearing loss between the left and the right sides, we found that left-sided tinnitus is more distressing than the right-sided one. Moreover, patients with the left-sided tinnitus had more complaints in the somatic subscale than the right-sided tinnitus patients. This could be attributed to neuroanatomic differences between the left and right parts of the auditory system.

Noise—either occupational or environmental noise pollution—is a major preventable cause of the hearing loss and therefore of chronic tinnitus. The consequences of noise exposure last for life, as the sensory auditory epithelium never regenerates. The price to pay for the noise exposure includes not only a limited ability to hear but also increased chance to develop chronic decompensated tinnitus, which separates the affected persons from normal social activities and significantly decreases the quality of life.

## 5. Conclusions

Our study demonstrates that the chain of events started by overexposure to noise not only induces the hearing loss but also tinnitus with its co-morbidities, finally resulting in a decreased quality of life. The degree of hearing impairment is reflected by the severity of tinnitus.

## Figures and Tables

**Table 1.** Scores of the TQ subscales measured in the 221 female and 220 male tinnitus patients.

**Subscale**	**Female**	**Male**
Emotional distress	9.49 ± 5.51	9.26 ± 5.34
Cognitive distress	6.04 ± 3.93	5.93 ± 3.85
Intrusiveness	9.68 ± 3.62	9.24 ± 3.61
Auditory perceptual difficulties	4.78 ± 3.60	4.23 ± 3.44
Sleep disturbances	3.35 ± 2.67	2.92 ± 2.50
Somatic complaints	1.98 ± 1.72	1.54 ± 1.68*

Given are the means ± standard deviations.

* p < 0.01 *vs*. female patients
